# Biological Activities of Natural Products III

**DOI:** 10.3390/molecules28124854

**Published:** 2023-06-19

**Authors:** Halina Maria Ekiert, Agnieszka Szopa

**Affiliations:** Chair and Department of Pharmaceutical Botany, Faculty of Pharmacy, Jagiellonian University, Collegium Medicum, 9 Medyczna Street, 30-688 Kraków, Poland

The search for natural products that display biological activity is invariably an attractive research area for scientific centers and teams from around the world. New natural products are eagerly awaited by the pharmaceutical, cosmetics and health food industries.

Products of natural origin exhibiting biological activity are most frequently obtained from higher plants. Additionally, endophytes, algae, fungi, lichens and animals (including insects) comprise valuable sources of these compounds.

In this the Special Issue, almost all papers focused on different species of higher plants as sources of biologically active compounds. Only one paper investigated the natural product of bees—propolis.

This Special Issue presents the results of experimental investigations and reviews prepared by scientific teams from Asia (China, India, Korea), Near East (Jordan, Palestine, Saudi Arabia), Africa (Egypt, Morocco), North America (USA), South America (Brazil), and several European countries (Bulgaria, Croatia, France, Germany, Poland and United Kingdom).

The results are presented via 15 original articles and 5 reviews. We are convinced that readers of *Molecules* will consider this Issue to be an important and valuable source of current knowledge about new natural products—the candidates for practical application in therapy, cosmetology, the health food industry and plant care.

It is conspicuous that this Issue contains as many as 4 papers (3 original papers and one review) that aim to highlight the use of natural extracts in skin and periodontal diseases. An additional group of especially impressive papers consists of 6 original contributions documenting the cytotoxic, antioxidant, anti-inflammatory and antiviral activity of plant extracts and propolis. Two original papers promote different types of plant in in vitro cultures as sources of bioactive compounds.

Kroma A. et al. [1] documented the beneficial effect of creams with ethanolic extract from the herb known as *Serratula coronata* in the treatment of psoriatic patients. The authors determined the primary group of components of this extract responsible for a significant reduction in the area of psoriatic plagues and improvement in skin conditions was probably ecdysteroids. The results were estimated using an ESI/HPLC-MS method.

Gaweł-Bęben K. et al. [2] presented the results of the application of pumpkin peel extracts from five *Cucurbita* spp. cultivars (*C. maxima*—2 cvs and *C. moschata*—3 cvs) prepared in different solvents (water, 50% propylene glycol, 20% ethanol) to skin care and dermatology. The water extracts showed the highest flavonoid contents, the greatest antioxidant potential (ABTS & DPPH scavenging assays), the highest SPF (sun protecting factor) and were not cytotoxic to human keratinocytes (up to a concentration of 1000 µg/mL). The investigated extracts contained large quantities of amino acids, fatty acids, flavonoids and phenolic acids beneficial to the treatment skin conditions. They also influenced the tyrosinase activity.

Dos Santos V. R. N. et al. [3] documented the reduction in symptoms of induced periodontitis in rats after the application of oleoresin (in the form of emulsion) from *Copaifera reticulata* (a tree species from Amazonia). Histopathological investigation and microcomputed tomography analysis documented notable degrees of inflammation inhibition. The primary components of oleoresin responsible for this effect may be β-caryophyllene (37.3%), β-bisabolene (14.5%) and β-bergamotene (9.0%).

The next few papers under review documented anticancer and cytotoxic activity of plant extracts and propolis, not to mention their antioxidant, anti-inflammatory and antiviral effects.

Noman O. et al. [4] studied the anticancer efficacy of chlorojanerin (a sesquiterpene lactone), isolated from ethanolic extracts of the aerial parts of *Cantaurothamnus maximus*, an endangered plant species growing in the southwestern mountains of the Arabian Peninsula. The anticancer activity of this compound was tested in vitro on 3 human cancer cell lines (lung, breast and colon adenocarcinomas A549, MCF-7, LoVo, respectively). The compound inhibited the proliferation of all tested cells in a dose-dependent manner, exhibiting the highest level of activity against A549 cells. The activity involved cell-cycle arrest and apoptotic cell death.

Dulović A. et al. [5] identified glucosinolates in different organs of *Sisimbrium officinale* and *Sisimbrium orientale* wild-growing in Croatia (8 and 6 compounds, respectively). The authors made use of the UHPLC-DAD-MS/MS method. Breakdown products of glucosinolates from *S. officinale* and isopropyl isothiocyanate were the major volatile compounds and were tested for their cytotoxic activity against three human cell lines (lung A549, bladder T24, and breast MDA-MB-231). Generally, the study showed similar activity for the above compounds, with isopropyl isothiocyanate exhibiting the highest potency (100 µg/mL after 72 h).

Wróbel-Biedrawa D. et al. [6] focused on the best conditions for rapanone extraction from leaves of a white-berry *Ardisia crenata* cultivar. Using three different extraction methods (heat reflux, shaking and ultrasound-assisted extraction), five solvents, different duration times and numbers of extractions were tested. Extraction with ethyl acetate or chloroform for 20 min. was the most efficient and cost-effective method. Additionally, the high cytotoxic activity exhibited by rapanone was confirmed (prostate DU145, PC3, thyroid FTC133, 8505C and colorectal Caco-2, HT29 cells). Rapanone revealed a more favorable safety profile than the embelin and doxorubicin investigated for comparison.

Soleman et al. [7] analyzed hexane extract of the leaves of *Annona glabra* (a tropical fruit tree native to Florida) using GC-MS. The extract was evaluated for its cytotoxic activity (against six human cancer cell lines) and antiviral activity (against HSV1 and HAV). The extract exhibited significant cytotoxic activity against Caco-2 and A-549 (colon cancer cell lines) and showed a promising protective and virucidal activity against HSV1. In silico molecular docking showed that limonene could be responsible for the strongest antiviral activity of the extract.

Oubihi et al. [8] concentrated their investigations on the chemical composition and anti-inflammatory and antioxidant effects of essential oil extracted from the aerial parts of *Thymus leptobotrys*, a plant endemic to Morocco. GC-MS analysis documented 31 compounds, with carvacrol serving as the primary compound. The essential oil exhibited strong anti-inflammatory activity in vivo (in rats) and strong antioxidant properties in vitro (DPPH & TEAC assays). The assessment of its potential toxicity via testing on mice indicated the safety of this essential oil.

Tyagi et al. [9] synthesized superparamagnetic iron oxide nanoparticles, which were coated with tamoxifen-conjugated bovine serum albumin. These conjugates were effective against breast cancer cell lines (MCF-7 and T47D). They were shown to be safe for use in drug delivery systems (according to the results of the acute toxicity study performed on rats). The authors suggested that these nanoparticles have potential as a drug delivery carrier system and for use in diagnostic tests, but clinical studies in humans must be performed before this begin.

Gogocz M. et al. [10] noted that the standard extraction method of propolis (heating in 70% ethanol) destroyed the complex compounds and caused the depletion of lipophilic compounds, both of which have biological activity. These authors proposed the use of cold extraction (100% ethanol, room temperature) and documented a significant anticancer activity of 75% and 80% cold propolis extract against two prostate cell lines in vitro (PC-3, DU-145—hormone-dependent and hormone-independent cell lines).

Two articles address the other activities of plant extract, namely, their antidiabetic, antioxidant, anti-obesity, and anti-steatotic activity.

Gościniak A. et al. [11] optimized the extraction conditions (% of methanol, time and temperature) for pomegranate flower extract (a raw material from Albania) with high antidiabetic effect, high antioxidant activity (DPPH test) and high content of pelargonidin- 3,5-glucoside, anthocyanin with antidiabetic activity. The authors sought to obtain the extract with the highest enzyme inhibition power (against α-amylase and α-glucosidase). They applied the Box-Benken method in the optimization of the process.

Oriquat et al. [12] investigated the anti-obesity and anti-steatotic effects of the flavonoid chrysin in an animal model (rats). Chrysin treatment results thoroughly documented that this compound has the potential to be a solid candidate for use in the treatment of obesity and associated nonalcoholic fatty liver disease. They showed multiple actions, exerting antioxidant-enhancing, glucose- and lipid-lowering, metabolic-enhancing, mitochondrial boosting and anti-inflammatory effects.

The next article under consideration explores the potential usefulness of synthetic derivatives of flavonoids in plant diseases.

Ma Y. et al. [13] synthesized a series of oxazinyl flavonoids on the basis of flavone (19 compounds) and tested them for antiviral activity against tobacco mosaic virus (TMV), as well as their capacity as anti-phytopathogenic fungus agents. Most of the compounds showed moderate-to-excellent antiviral activities. Some compounds possessed a better antiviral activity than ribavirin, apigenin and ningnanmycin. The oxazinyl flavonoids exhibited broad spectrum fungicidal activity (6 kinds of pathogenic plant fungi were tested) and were proposed for use as a “green pesticide” with high efficiency and low toxicity.

Two original articles present biotechnological studies and propose the biomass obtained from in vitro cultures to be a rich potential source of bioactive natural products.

Krasteva G. et al. [14] reported the first study into metabolite profiles, antioxidant activity and genetic variations of *Gardenia jasminoides* in in vitro cultures with different levels of differentiation in terms of shoot cultures, callus and suspension cultures. Shoots and leaves (from plants grown in vivo) produced more individual compounds and their higher amounts than callus and suspension cultures. Antioxidant activity (DPPH, FRAP, TEAC & CUPRAC assays) and total phenolics were also higher for shoot and leaf extracts. All in vitro cultures showed a high genetic variability.

Kwiecień I. et al. [15] established agitated and bioreactor (PlantForm bioreactors) microshoot cultures of three *Hypericum perforatum* cvs—Elixir, Helos and Topas—and tested different concentrations of cytokinin (BAP) and auxin (NAA), in addition to different durations of growth cycles (5 and 4 weeks, respectively), achieving large amounts of phenolic acids, flavonoids and catechins (505, 2386 and 712 mg%, respectively—Helos cv.). The extracts of biomass showed high or moderate antioxidant activity (3 in vitro tests), high activity against G(+) bacterial strains and strong antifungal activity. In the precursor feeding experiments (phenylalanine—1 g/L), the total content of metabolites was enhanced 2.33-, 1.73- and 1.33-fold, respectively, and reached the max. for Elixir cv. (4.48 g%).

Five review articles are dedicated to different scientific problems.

Luo L. et al. [16] presented the available investigations (for a period of 45 years) connected with the taxonomy, chemical composition and pharmacological activity of *Polygonatum* species. The genus *Polygonatum* comprises 79 plant species, of which about 39 species grow in China. The principal metabolites of this taxon include steroidal saponins (124 compounds), flavonoids (68), triterpenoid saponins (16) and alkaloids (14). Rhizome extracts exhibited antioxidant, anti-fatigue, anti-inflammatory, anti-hypoglycemic and immunological activities. According to the Chinese Pharmacopoeia, wine steaming and wine stewing are the best extraction conditions for realizing pharmacological activity of the extracts.

Jafernik K. et al. [17] reviewed the current state of knowledge on chemical composition and biological activity of *Schisandra henryi*, a plant species endemic to Yunnan Province (China) with connections to the famous phytotherapeutic *Schisandra chinensis*. The primary groups of metabolites in *S. henryi* include different types of lignans (dibenzocyclooctadiene, aryltetralin and dibenzylbutane), phenolic acids, flavonoids, triterpenoids and nortriterpenoids. The authors proposed the biomass from in vitro cultures established by them as a solid source of bioactive compounds, especially some dibenzocyclooctadiene lignans, phenolic acids and flavonoids (max. total amounts of 873.71, 840.89 and 421.98 mg%, respectively). Additionally, they reviewed the different directions of biological activity of dibenzocyclooctadiene lignans, which are the main metabolites in *S. chinensis* and also in *S. henryi*.

Kopalli et al. [18] discussed published reports of studies on the possible role of natural product-derived biomolecules in beneficial effect in atopic dermatitis and other related skin disorders, primarily focusing on SOCS protein expression and JAK/STAT signaling. These biomolecules include resveratrol, astragalin, diosmetin, curcumin, luteolin and quercetin.

Neba Ambe G. N. N. et al. [19] reviewed literature data suggesting that age-related diseases and metabolic syndrome might be caused by dysregulated and dampened clocks. The authors highlighted dietary compounds that positively affect the maintenance of a circadian clock and promoted nobiletin (polymethoxyflavone) as a promising candidate with potential chronotherapeutic action.

Mączka et al. [20] collected recent studies on the antimicrobial properties of linalool, its mechanism of action on cells and involvement in detoxification processes. Its mechanism of action on microbes is multifaceted. Linalool is a good candidate for a combination therapy with antibiotics.

Summing up, acknowledging the excellent scientific teams that have contributed to this issue, we are convinced that the readers of *Molecules* will find this third part of the Special Issue, dedicated to biological activities of natural products, to be both attractive and inspiring.
